# Immunomodulation of the NLRP3 Inflammasome in Atherosclerosis, Coronary Artery Disease, and Acute Myocardial Infarction

**DOI:** 10.1007/s12265-020-10049-w

**Published:** 2020-07-09

**Authors:** Max J. M. Silvis, Evelyne J. Demkes, Aernoud T. L. Fiolet, Mirthe Dekker, Lena Bosch, Gerardus P. J. van Hout, Leo Timmers, Dominique P. V. de Kleijn

**Affiliations:** 1 Department of Cardiology, University Medical Center Utrecht, Heidelberglaan 100, 3508 GA Utrecht, The Netherlands; 2 Department of Experimental Cardiology, University Medical Center Utrecht, Utrecht, The Netherlands; 3 Department of Vascular Surgery, University Medical Centre Utrecht, Utrecht, The Netherlands; 4 Department of Cardiology, Amsterdam University Medical Centre, Amsterdam, The Netherlands; 5 Department of Cardiology, St. Antonius Hospital, Nieuwegein, The Netherlands

**Keywords:** NLRP3 inflammasome, Atherosclerosis, Coronary artery disease, Interleukin-1β, Interleukin-18, Ischemia-reperfusion injury, Myocardial infarction

## Abstract

Cardiovascular disease (CVD) remains the leading cause of mortality and morbidity worldwide. Atherosclerosis is responsible for the majority of cardiovascular disorders with inflammation as one of its driving processes. The nucleotide-binding oligomerization domain-like receptor family pyrin domain containing 3 (NLRP3) inflammasome, responsible for the release of the pro-inflammatory cytokines, interleukin-1β (IL-1β), and interleukin-18 (IL-18), has been studied extensively and showed to play a pivotal role in the progression of atherosclerosis, coronary artery disease (CAD), and myocardial ischemia reperfusion (I/R) injury. Both the NLRP3 inflammasome and its downstream cytokines, IL-1ß and IL-18, could therefore be promising targets in cardiovascular disease. This review summarizes the role of the NLRP3 inflammasome in atherosclerosis, CAD, and myocardial I/R injury. Furthermore, the current therapeutic approaches targeting the NLRP3 inflammasome and its downstream signaling cascade in atherosclerosis, CAD, and myocardial I/R injury are discussed.

## Introduction

Cardiovascular disease (CVD) remains the leading cause of mortality and morbidity worldwide, despite major improvements in treatment and outcomes. CVD is a group of diseases that effects the heart and blood vessels including coronary artery disease (CAD), cerebrovascular disease, and peripheral artery disease [1, 2]. CAD is characterized by the accumulation of atherosclerotic plaque in the coronary arteries and its major clinical consequence is an acute myocardial infarction (MI) [3].

Atherosclerosis is a progressive disease that is characterized by the accumulation of lipids, inflammatory cells, and fibrous elements in arterial plaques. It is the driving force in CAD and interventions that aim to influence the development, progression, and stability of atherosclerotic lesions are of pivotal importance. Controlling risk factors that lead to disease progression, such as smoking, physical inactivity, poor-quality diet, high blood pressure, and diabetes mellitus, leads to the reduction of atherosclerosis related morbidity and mortality [4]. Management of dyslipidemia is the cornerstone in primary and secondary prevention of atherosclerotic disease, with a 20% relative risk reduction of major cardiovascular events per mmol/L low-density lipoprotein (LDL)-cholesterol reduction [5]. However, despite lifestyle optimization and medical treatment strategies, major cardiovascular events still occur in a substantial proportion of the population. This issue is commonly described as the problem of “residual risk”.

Such residual risk for developing a cardiovascular event may reflect aspects of atherogenesis, such as specific inflammatory pathways, which are not targeted by the current treatment strategies [6, 7].

Inflammation is a key mechanism in atherogenesis and is described as a process by which the body responds to harmful stimuli. It is mediated by components of the innate and adaptive immune system and involves a complex interplay of circulating and resident cells, like neutrophils and macrophages that lead to plaque progression, destabilization, and subsequent cardiovascular events, such as MI [6].

### Inflammasome

In 2002, the term “inflammasome” was first used for a multiprotein complex that was involved in the activation of caspase-1 and subsequently resulted in interleukin-1ß (IL-1ß) activation and release [8]. IL-1ß is a well-characterized pro-inflammatory cytokine that plays a central role in many inflammatory diseases. Inflammasomes are intracellular protein complexes that are merged after pattern recognition receptors (PRRs) bind danger-associated molecular patterns (DAMPs) or pathogen-associated molecular patterns (PAMPs). DAMPs can be defined as endogenous molecules released after damage or stress, such as extracellular adenosine triphosphate (ATP) and cholesterol crystals. PAMPs are from exogenous origin, such as toxins that are expressed by bacteria and viruses [9, 10].

Several inflammasomes have been identified as follows: the nucleotide-binding oligomerization domain (NOD); leucine-rich repeat (LRR)-containing protein (NLR) family members, NLRP1, NLRP3, NLRC4; and the proteins absent in melanoma 2 (AIM2) and pyrin inflammasome. Each inflammasome is activated by different stimuli with the NLRP3 inflammasome as the best characterized inflammasome [9, 11].

### The NLRP3 Inflammasome and its Components

The NLRP3 inflammasome consist of three components: (1) NLRP3, (2) Apoptosis speck-like protein (ASC), and (3) caspase-1.

**Fig. 1:**
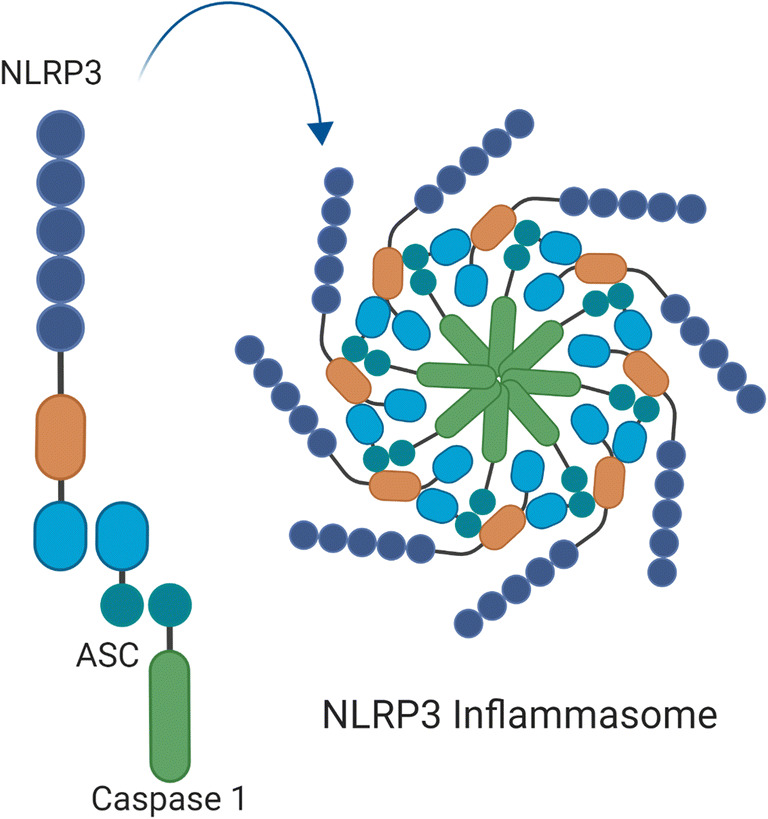
NLRP3 inflammasome components. The NLRP3 inflammasome consist of three components: NLRP3, ASC, and caspase-1. NLRP3 protein contains three domains: NACHT, a central nucleotide domain; LRR, C-terminal leucine-rich repeats; and PYD, pyrin domain. ASC contains two interaction domains: pyrin domain (PYD) and caspase-recruitment domain (CARD). Caspase-1 contains a CARD and catalytic domain. Figure was created with Biorender.com

The innate immune receptor, NLRP3, contains three domains: NACHT, a central nucleotide domain LRR; C-terminal leucine-rich repeats; and PYD, pyrin domain. The adaptor protein, ASC, contains two interaction domains: pyrin domain (PYD) and caspase recruitment domain (CARD). Upon activation via DAMPs or PAMPs, NLRP3 recruits the adaptor protein ASC through PYD-PYD interactions. Caspase-1 is a cysteine protease and contains a CARD and catalytic domain. ASC is able to bind to caspase-1 through CARD-CARD interactions [9, 11]. The activation of this complex results in the activation of caspase-1, a proteolytic enzyme that in turn is responsible for the activation of IL-1β and IL-18 [12]. In addition, caspase-1 induces a proinflammatory form of regulated cell death termed pyroptosis [13] (Fig. 1).

### NLRP3 Inflammasome Priming and Activation

Activation of the NLRP3 inflammasome in immune cells usually requires two steps: priming and activation. Priming leads to the activation of the transcription factor nuclear factor κB (NF-κB) and upregulation of inflammasome components and pro-IL-1β. It is dependent on the stimulation of PRRs, such as the membrane Toll-like receptors (TLRs) [10, 14–17]. Activated cytokines, such as tumor necrosis factor (TNF) and IL-1ß, are also known to provide the priming signal [10].

After formation of the essential proteins, final activation of the NLRP3 inflammasome occurs through oligomerization of the three components.

This final activation occurs due to a variety of DAMPs, including increased extracellular ATP, particulates, and crystals. These triggers induce several upstream signaling events like potassium (K^+^) efflux and mitochondrial reactive oxygen species production, leading to the final activation [18, 19].

Emerging evidence is showing an important role of NLRP3 inflammasome-mediated ignaling in atherogenesis, identifying the NLRP3 inflammasome as a potential target in patients with established atherosclerotic CAD [17, 19, 20]. Next to this, the NLRP3 inflammasome, caspase-1 induced pyroptosis, and inflammation mediated by IL-1ß and IL-18 have shown to be involved in ischemia reperfusion (I/R) injury after an acute MI [15, 17].

This review not only summarizes the role of the NLRP3 inflammasome in atherosclerosis but also discusses its role in CAD and myocardial I/R injury after an acute MI, thereby providing an in depth overview of its dual role in coronary artery disease. Furthermore, the current (pre)clinical therapeutic approaches targeting the NLRP3 inflammasome and its downstream signaling cascade in atherosclerosis, CAD, and myocardial I/R injury are discussed.

## Atherosclerosis

### Inflammation in Atherosclerosis

Inflammation in the early phase of atherosclerosis is thought to be the consequence of endothelial injury with subsequent production of adhesion molecules that can capture inflammatory cells that migrate into the arterial wall. Monocytes differentiate into macrophages and can phagocytose the LDL particles to form foam cells [21]. Foam cells contribute to the accumulation of the atheroma in the plaque and secrete pro-inflammatory cytokines, such as TNF-α and IL-1ß. Together with inflammatory cells, these cytokines enhance the development of early atherosclerotic plaques that are called fatty streaks. Infiltration of more inflammatory cells and smooth muscle cells attracted by the cytokines leads to more complex lesions with a fibrous cap [22]. With increasing plaque size, neovascularization can lead to hemorrhage in the plaque enhancing influx of inflammatory cells and lipids. Cytokines also increase the production and activation of matrix metalloproteases that can degrade the collagen fibers in the fibrous cap. Rupture of the fibrous cap and subsequent thrombosis may occlude the artery and leads to major cardiovascular complications like acute MI [23, 24].

Since inflammation is a key process in the initiation, and the progression of atherosclerosis to unstable atherosclerotic plaques prone to rupture, inflammatory pathways are potential targets in the treatment of atherosclerosis on top of lipid-lowering. The role of inflammation and the NLRP3 inflammasome in atherosclerosis has been studied extensively in atherosclerotic mouse models.

### Mouse Models of Atherosclerosis

The most common mouse models used are the Apolipoprotein E deficient (ApoE−/−) and the LDL receptor-deficient (LDLr−/−) mice, both of which develop hypercholesterolemia.

ApoE−/− mice naturally develop complex lesions on a normal diet but this process can be accelerated by a fat and cholesterol enriched diet. The ApoE−/− model shows a different lipoprotein phenotype compared with humans since the majority of plasma cholesterol is carried by VLDL and chylomicrons particles. In humans, the cholesterol is mainly transported by LDL. When LDLr−/− mice are placed on a high-fat, high-cholesterol diet, this leads to an accumulation of (V)LDL that is richer in triglyceride and apoB100 than in ApoE−/− mice, but when LDL−/− mice are on a normal diet, only small lesions develop [25, 26].

### NLRP3 Inflammasome in Atherosclerosis and the Role of Cholesterol Crystals

Mouse studies were used to investigate the effects of the NLRP3 inflammasome’s downstream cytokines, IL-1β, and IL-18 on atherosclerosis in the early 2000s. Kirii et al. showed that, in mice lacking both apolipoprotein E (ApoE) and IL-1β, the lack of IL-1β decreased aortic atherosclerotic lesion size, possibly through increased expression of vascular cell adhesion molecule-1 and monocyte chemotactic protein-1 [27]. Mallat et al. observed that endogenous inhibition of IL-18 in mice prevented early lesion development and changed lesion composition into a more stable plaque phenotype with a decrease in macrophages, T-cells, cell death, and lipid content and an increase in smooth muscle cell and collagen content [28].

Nearly 10 years later, Duewell et al. showed the importance of the NLRP3 inflammasome in the development and progression of atherosclerosis [29]. First, they revealed that cholesterol crystals (CCs) in early atherosclerotic lesions coincide with the presence of inflammatory cells. Second, when CCs were injected intraperitoneally in mice, it induced a profound inflammatory response. This was impaired in mice that were deficient for the NLRP3 inflammasome components.

Furthermore, CCs have been shown to strongly activate the NLRP3 inflammasome in mouse and human macrophages [29, 30].

The importance of the NLRP3 inflammasome was also studied in atherosclerosis-prone, LDLr−/− mice on a high cholesterol diet, lacking either NLRP3, ASC, or IL-1. Mice deficient for NLRP3, ASC, or IL-1α/β showed a significant reduction of atherosclerotic lesion size and significantly lower levels of IL-18 [31].

In contrast, a recent study suggested that IL-1ß also has beneficial effects in late-stage atherosclerosis in an ApoE−/− mouse model. Mice with advanced atherosclerosis were treated with anti-IL-1ß and this treatment induced a marked reduction in smooth muscle cells and collagen content, accompanied by an increased macrophage count in the fibrous cap [32]. These results indicate that IL-1β can act as a double-edged sword. Detrimental at the initiation and progression of atherosclerosis and beneficial in a later and advanced stage.

Furthermore, Menu et al. reported no difference in the progression of atherosclerosis, infiltration of plaques, or plaque stability in ApoE−/− mice that were deficient for either NLRP3, ASC, or caspase-1 compared with wildtype mice [33]. This was explained by the fact that in the mouse model used (ApoE−/−), IL-1α mostly contributes to the development of atherosclerosis. Since IL-1α does not need NLRP3 activation to be generated, missing its key components would not affect atherosclerosis. However, other studies also using ApoE−/− mice showed that caspase-1 deficiency decreased atherosclerotic plaque size [34, 35].

### Activation of NLRP3

Numerous signals, present in the atherosclerotic lesions, are known to activate the NLRP3 inflammasome. As mentioned before, CCs are potent enhancers of the NLRP3 inflammasome in macrophages, and additionally, oxidized LDL (oxLDL) was also shown to play a role in NLRP3 inflammasome activation [36, 37].

Since atherosclerotic plaques contain a large amount of CCs and oxLDL, this may result in a vicious circle of NLRP3 inflammasome priming and activation in macrophages, with attraction of more macrophages and NLRP3 activation.

**Fig. 2:**
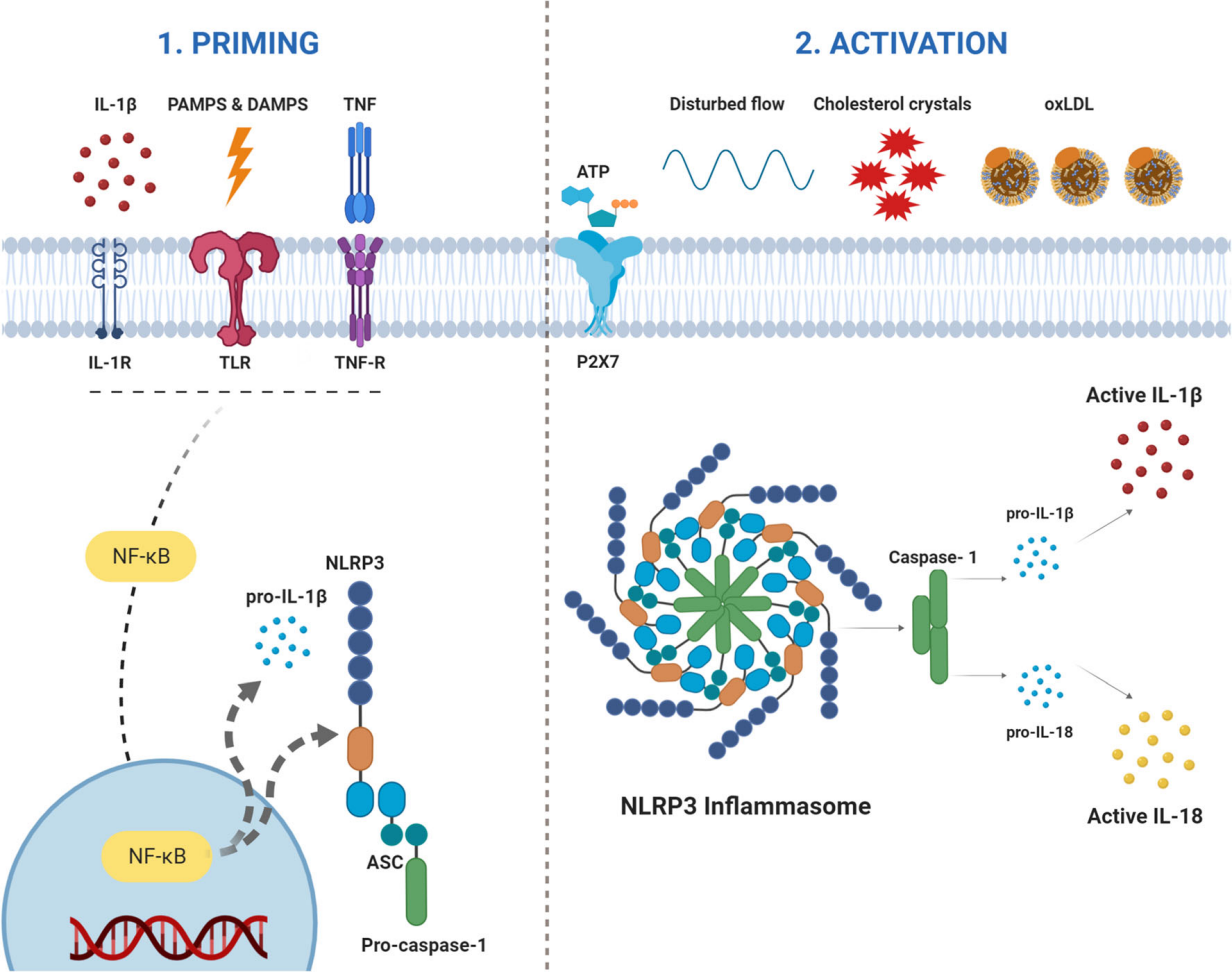
Inflammasome activation in atherosclerosis requires two steps; priming and activation. PRRs, such as TLRs, get stimulated by PAMPS & DAMPS leading to upregulation of the inflammasome components. Signals present in the atherosclerotic lesions (cholesterol crystals and oxidized LDL, disturbed flow) lead to activation of the inflammasome (activation). The NLRP3 inflammasome activates caspase-1 that in turn is responsible for the activation of interleukin-1β (IL-1β) and interleukin-18 (IL-18). Figure was created with Biorender.com

Next to CCs and oxLDL, calcium phosphate crystals also contribute to the progression of atherosclerosis and the instability of plaques through activation of the NLRP3 inflammasome [37]. Disturbance in blood flow, hypoxia, and acidosis has also shown the ability to augment NLRP3 inflammasome activation [38–40] (Fig. 2).

## Coronary Artery Disease

CAD is characterized by accumulation of atherosclerotic plaque in the coronary arteries. It is a progressive, chronic disease and the aim of current treatment is to achieve stabilization of the disease. Progressive atherogenesis can culminate in acute plaque rupture or endothelial erosion with subsequent thrombosis leading to coronary occlusion and acute MI [3].

### Inflammation in CAD

Both local and systemic inflammations are believed to have significant influence on the progression of stable CAD to plaque instability and rupture [41]. Abundant epidemiologic data supports a link between systemic inflammation and cardiovascular outcomes. A higher baseline C-reactive protein (CRP) is associated with the risk for future cardiovascular events, such as stroke and MI and a reduction of risk appears to be directly related to a reduction in CRP [42]. Meta-analysis including more than 160.000 patients shows a continuous association of CRP concentration with the risk of CVD [43].

Interleukin-6 (IL-6) is a more upstream inflammatory marker of hsCRP and downstream of the NLRP3 inflammasome, IL-1ß, and IL-18. IL-6 is also strongly associated with future risk of major cardiovascular events [44, 45]. Mendelian randomization studies support a potential causal role of IL-6 in atherothrombosis but data directly relating IL-6 reduction to vascular event reduction is scarce [46].

IL-1β and IL-18 induce the production of IL-6 which further supports the idea that the NLRP3-inflammasome could act as a major target for cardiovascular prevention [37, 47].

### NLRP3 Inflammasome and CAD

Several studies focusing on patients with CAD and its relation to NLRP3 inflammasome activation, IL-1β, and IL-18 have been reported. IL-1β was shown to be present in luminal and adventitial vessel endothelial cells and macrophages of coronary arteries [48]. Serum IL-18 levels also showed to be associated with cardiovascular death in patients with CAD [49].

An association between the NLRP3 inflammasome and patients with established CAD was published by Zheng et al. [50]. The authors demonstrated that aortic NLRP3 expression, measured with immunohistochemistry, highly correlated with the severity of coronary atherosclerosis (measured by the Gensini score), in patients undergoing coronary artery bypass graft surgery (*r* = 0.673, *P =* <0.05).

Comparing the levels of NLRP3 inflammasome in PBMCs and plasma IL-1β and IL-18 levels between patients with and without CAD showed that all levels were higher in patients with CAD supporting the association between CAD and NLRP3 inflammasome activation [51].

These observations on the association of inflammation, the levels of downstream cytokines of the NLRP3-inflammasome, and the severity of atherosclerotic disease led to the development of trials investigating anti-inflammatory therapies.

## Clinical Trials that Aim to Reduce Cardiovascular Events by Targeting Inflammation Related to the NLRP3 Inflammasome in Patients with CAD

Clinical trials with selective NLRP3 inflammasome inhibitors have not been performed but multiple studies targeting inflammatory pathways that are related to the NLRP3 inflammasome are either completed or are ongoing (Table 1).

**Table 1:** Clinical trials targeting inflammation related to the NLRP3 inflammasome

Name of the study	Published	Indication	Sample size	Intervention	Outcome	Results	Ref
LoDoCo: Low-Dose Colchicine for Secondary Prevention of Cardiovascular Disease	2013	Stable coronary artery disease	532	Colchicine 0.5 mg/d (*n* = 282) vs. no colchicine (*n* = 250)	Composite incidence of ACS, OHCA, ischemic Stroke	Colchicine 5.3% primary outcome No colchicine 16.0% primary outcome	[52]
COLCOT: Colchicine Cardiovascular Outcomes Trial	2019	Recent myocardial infarction (within 30 days after MI)	4745	Colchicine 0.5 mg/d (*n* = 2366) vs. placebo (*n* = 2379)	Composite of cardiovascular death, resuscitated cardiac arrest, MI, stroke, urgent hospitalization for angina leading to coronary revascularization	Colchicine 5.5% primary outcome Placebo 7.1% primary outcome	[53]
CANTOS: Canakinumab Anti-inflammatory Thrombosis Outcome Study	2017	History of myocardial infarction and hsCRP of 2 mg or more	10,061	Canakinumab 50 mg/3 months (*n* = 2170) 150 mg/3 months (*n* = 2284) 300 mg/3 months (*n* = 2263) Placebo (*n* = 3344)	Nonfatal myocardial infarction, nonfatal stroke, cardiovascular death	Canakinumab 150 mg/3 months incidence primary endpoint 3.86 events per 100 person-years Placebo incidence primary endpoint 4.50 events per 100 person-years	[54]
LoDoCo2: Low-dose Colchicine for Secondary Prevention of Cardiovascular Disease	N/A	Stable coronary artery disease	5522	Colchicine 0.5 mg/d vs. Placebo	Composite of cardiovascular death, myocardial infarction, ischemic stroke, ischemia-driven revascularization	Expected to publish results in 2020	[55]
COACS: Colchicine for Acute Coronary Syndromes	N/A	Acute Coronary Syndrome	500	Colchicine 0.5 mg/d vs. Placebo	Overall mortality, new acute coronary syndrome, ischemic stroke	Unknown	[56]
CLEAR-SYNERGY: Colchicine and Sprionolactone in Patients with STEMI/SYNERGY stent registry	N/A	STEMI	4000	Colchicine 1 mg/day and/or spironolactone 24 mg/day and/or placebo and/or SYNERGY stent (2 × 2 factorial design)	Cardiovascular death, recurrent myocardial infarction or stroke in the colchicine comparison	Estimated completion 2021	[57]
VCU-ART/VCU-ART(2): Virginia Commonwealth University Anakinra Remodeling Trial	2010/2013	ST-Elevation Myocardial Infarction	10/30	Anakinra 100 mg/d (*n* = 16) vs. Placebo (*n* = 19)	Difference in interval change in the LV end-systolic volume index (LVESVi)	Trend towards reduced incidence of adverse remodeling/No observable benefits in LV remodeling or function	[58, 59]

## Colchicine

The first trial that demonstrated that targeting the inflammasome/inflammation can reduce cardiovascular events in patients with CAD was a study with colchicine.

Colchicine is an alkaloid, derived from the plant *Colchicum autumnale*. The drug possesses broad anti-inflammatory effects. Current indications for colchicine are (pseudo)gout, pericarditis, and Familial Mediterranean Fever (FMF). Its primary mechanism of action is microtubule inhibition which results in downregulation of various inflammatory pathways including the NLRP3 inflammasome [60].

Martinon et al. were the first to show that colchicine could act on the inflammasome [61]. Cells from the differentiated monocytic cell line THP1 were used and stimulated with inflammasome activators (monosodium urate (MSU), calcium pyrophosphate dehydrate (CPPD) or ATP) in the presence or absence of colchicine. The presence of colchicine completely blocked the processing of IL-1β.

After this, multiple studies have confirmed the inhibitory effects of colchicine on the NLRP3 inflammasome. Colchicine leads to the inhibition of the NLRP3 inflammasome in at least four mechanisms: (1) inhibition of the gene, resulting in Pyrin inhibition, (2) inhibition of inflammasome cytoplasmic co-localization due to tubulin interference, (3) direct caspase-1 blockage, and (4) inhibition of P2X7-mediated pore formation, resulting in decreased K+ efflux [62].

### LoDoCo

The LoDoCo study was a randomized, observer-blinded endpoint study where 532 patients with stable CAD were assigned to colchicine 0.5 mg or no colchicine and were followed for a median of 3 years. The primary end point, which was a composite of cardiovascular endpoints, occurred in 5.3% of the group that received colchicine compared with 16% of the group assigned to no colchicine (hazard ratio: 0.33; 95% confidence interval [CI] 0.18 to 0.59; *P* = <0.001) [52].

These promising results are currently being evaluated in the LoDoCo2 study, which will provide information on the efficacy and safety of daily administration of 0.5 mg colchicine, compared with placebo, for the secondary prevention in patients with stable CAD in a total of 5522 patients [55].

### COLCOT

Colchicine was also studied in the COLTOT trial. The COLCOT trial randomized a total of 4745 patients to low-dose colchicine of 0.5 mg once daily or placebo within 30 days after MI. The primary end point occurred in 5.5% of the patients receiving colchicine and in 7.1% of the patients receiving placebo (hazard ratio, 0.77; 95% confidence interval [CI], 0.61 to 0.96; *P* = 0.02.) The result was predominantly due to a lower incidence of stroke and urgent hospitalizations for angina leading to coronary revascularization [53]. The incidence of pneumoniae was also significantly higher in patients treated with colchicine compared with placebo as well. (0.9% vs. 0.4%, *P* = 0.03).

Two other ongoing trials that are investigating colchicine are the Colchicine for Acute Coronary Syndromes trial (COACS) and Colchicine and Spironolactone in patients with ST Elevation Myocardial Infarction (STEMI/Synergy stent Registry) (CLEAR-SYNERGY) [56, 57]. These studies will provide insight on the ability of colchicine to prevent new cardiovascular events in patients post ACS/STEMI.

## Canakinumab

### CANTOS

The Canakinumab Anti-inflammatory Thrombosis Outcome Study (CANTOS) was a large, randomized, double-blind trial that tested canakinumab in 10,061 patients with previous MI and a hsCRP level of 2 mg or more per liter. Canakinumab is a human monoclonal antibody targeting IL-1β. The study showed that 150 mg of canakinumab led to a significantly lower rate of recurrent cardiovascular events than placebo, and this reduction was independent of LDL level lowering (hazard ratio vs. placebo, 0.83; 95% CI, 0.73 to 0.95; *P* = 0.005) [54]. These promising observations were accompanied by a higher incidence of fatal infections compared with placebo but there was no significant difference in all-cause mortality.

It was the first time that direct targeting of inflammation by a selective IL-1β inhibitor, an end product of inflammasome activation, resulted in the reduction of cardiovascular events. These effects were related to a reduction of hsCRP and IL-6 levels [54, 63].

Although promising, a substantial part of participants remained at considerable risk for recurrent CV events and this risk was related to baseline IL-6 levels or IL-18 levels. Canakinumab’s selective inhibition of IL-1β did not reduce IL-18 levels [64]. Since activation of the inflammasome is leading to the activation of both IL-1β and IL-18, these observations encourage further development of trials that investigate pharmacologic agents that target the upstream NLRP3 inflammasome. Furthermore, canakinumab’s cardiovascular registration has been halted by Novartis, so for the time being, it will not become available in preventative cardiovascular medicine.

Trials investigating colchicine and the CANTOS trial are encouraging as they show beneficial effects of anti-inflammatory treatment in the prevention of cardiovascular events. Colchicine is known to be a non-selective inhibitor of the NLRP3 inflammasome and in CANTOS a selective IL-1ß inhibitor was studied.

Future research investigating the effects of selective NLRP3 inflammasome inhibition should also further evaluate the risk of adverse effects of treatment, like fatal infections and pneumoniae as was seen in CANTOS and COLCOT respectively.

## Myocardial Ischemia Reperfusion Injury

During an acute MI, the area of the myocardium that is ischemic is known as the area at risk (AAR). If the coronary artery remains occluded, most of the AAR becomes necrotic. To prevent myocardial tissue loss, reestablishing blood supply to the AAR by coronary revascularization is the cornerstone of current treatment [65].

Albeit revascularization salvages substantial myocardial tissue, experimental data suggests that return of perfusion (reperfusion) after MI also induces paradoxical additional myocardial damage. This is called ischemia/reperfusion (I/R injury) and is the result of a rapid correction of intracellular acidosis that leads to intracellular calcium overload, mitochondrial dysfunction, and oxidative stress. This hyper acute response is followed by a disproportional sterile inflammatory reaction and leads to expansion of infarct and deterioration of cardiac function [66, 67].

After myocardial tissue is injured, cellular debris and several intracellular and extracellular stimuli that are expressed after disruption of cell homeostasis can act as DAMPs leading to priming and triggering of the NLRP3 inflammasome in cardiomyocytes, leukocytes, fibroblasts, and endothelial cells [15, 68].

Kawaguchi et al. were the first to directly show an association between the NLRP3 inflammasome and MI when their work showed that ASC was markedly expressed in myocardial tissue that was obtained from patients that died after an acute MI [69]. In addition, wild type mice were subjected to myocardial I/R injury and had significant larger myocardial infarct size compared with mice deficient for ASC and caspase-1. This observation was accompanied by an increased inflammatory cell infiltration and cytokine expression (IL-1β, IL-6, IL-18, IL-10, MCP-1, TNF-α, and IFN-ϒ).

**Fig. 3:**
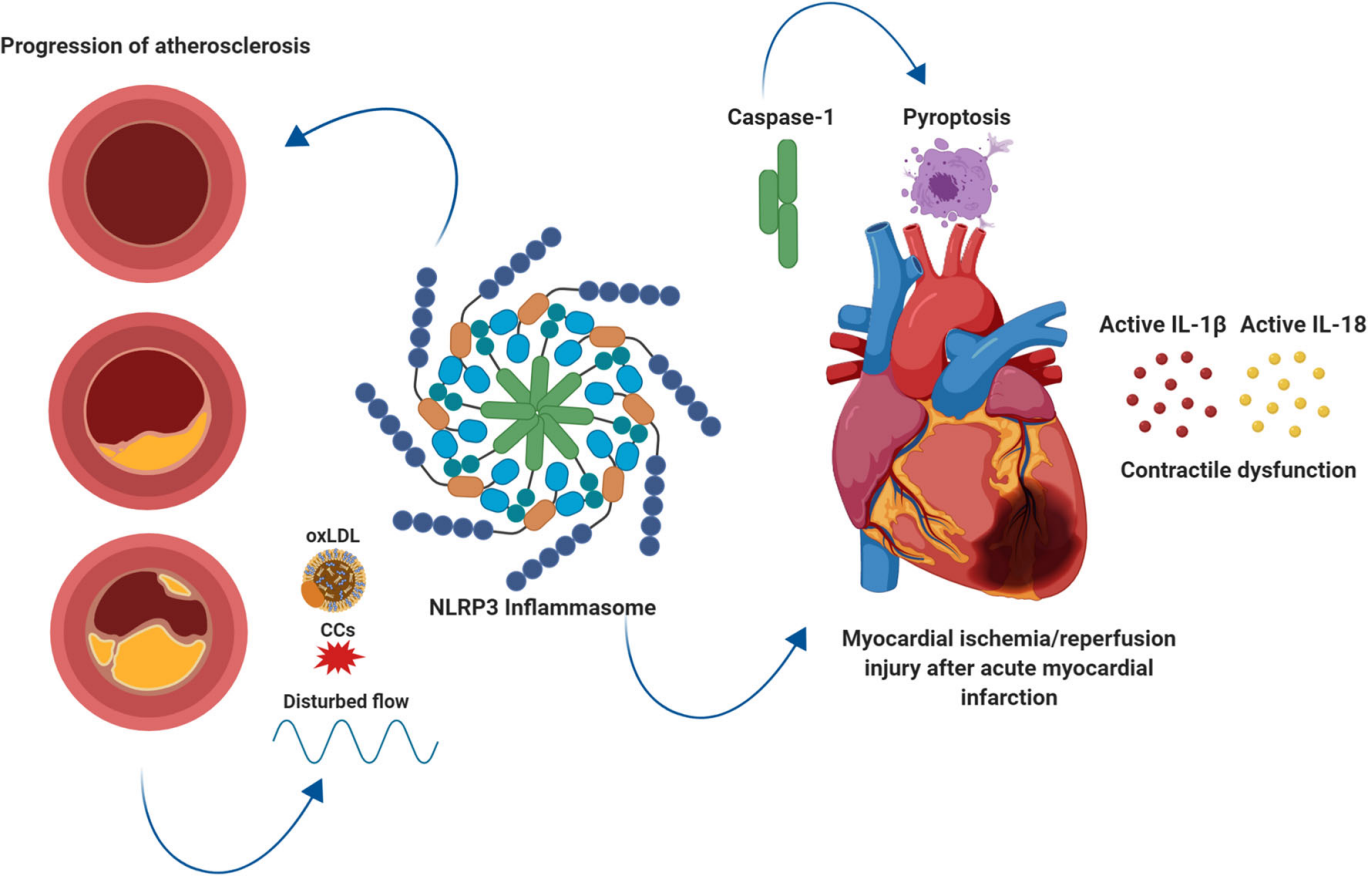
Central illustration: the NLRP3 inflammasome plays a dual role in coronary artery disease. (1) Progression of atherosclerosis. (2) Myocardial ischemia/reperfusion injury. Figure was created with Biorender.com

These results were further supported by other preclinical studies that observed a central role of the NLRP3 inflammasome and its components in acute MI. Mezzaroma et al. showed that ASC and caspase-1 were activated in acute MI in mice and that not only circulating inflammatory cells but also cardiomyocytes contain the structural components of the NLRP3 inflammasome [70]. Whereas activation of the inflammasome in circulating cells leads to IL-1β and IL-18 release, activation in cardiomyocytes leads to caspase-1 mediated pyroptosis. Furthermore, silencing RNA for NLRP3 or P2X7 (a receptor involved in inflammasome activation) in this mouse model limited infarct size after acute MI. Sandanger et al. induced MI in adult mice and Wistar rats and showed increased expression of NLRP3, IL-1β and IL-18 mRNA [71]. In an ex vivo Langendorff model of I/R injury, mice deficient for NLRP3 showed smaller infarcts. This was, however, not observed in hearts deficient for ASC. A possible explanation given for this discrepancy is that ASC also acts as an adaptor protein in other inflammasomes (Fig. 3, central illustration).

One of the first preclinical studies showing pharmacological inhibition could have beneficial effects on infarct size, and LV function following acute MI was performed with the compound BAY 11-7082. BAY 11-7082 is a NF-κB inhibitor. NF-κB is involved in the priming step of inflammasome activation and regulates the expression of different NLRP3 inflammasome components. BAY 11-7082 was also shown to be a direct inhibitor of the NLRP3 inflammasome by inhibiting the ATPase activity of NLRP3 [72]. Kim et al. showed that pre-treatment with BAY 11-7082, 30 min before infarct induction resulted in a significant reduction of infarct size and the preservation of cardiac function in rats [73]. This finding was further supported by other experimental studies in mice. Like mentioned before, Mezzaroma et al. showed that silencing RNA for NLRP3 or P2X7 limited infarct size. The same was seen with pharmacologic inhibition of P2X7 [70].

Marchetti et al. showed that a compound named 16673-34-0, which is a NLRP3 inflammasome inhibitor derived from glyburide, is acting on cardiomyocytes and preventing NLRP3 inflammasome oligomerization, could also reduce infarct size in a mouse model of I/R injury [74]. Next to reducing infarct size, the compound also preserved cardiac function when given in a single or repeated dose. These beneficial effects were also observed in a model of permanent occlusion [75].

OLT1177 (dapansutrile), a β-sulfonyl nitrile molecule and a selective inhibitor of the NLRP3 inflammasome, reduced infarct size in a dose-dependent manner in a murine model of myocardial I/R injury [76, 77]. OLT1177 works by inhibiting the ATPase activity of the NLRP3 protein. The effect of delayed administration was also studied and a cardio protective effect was observed when the compound was given 60 min after reperfusion. This effect was comparable with administration directly following reperfusion. Prolonged administration (120 and 180 min after reperfusion) did not result in a reduction of infarct size.

Colchicine, a drug with broad anti-inflammatory effects including inhibitory effects on the NLRP3 inflammasome also showed to be beneficial in experimental acute MI [62]. Multiple groups showed that administration of colchicine could result in a significant reduction of infarct size and improved adverse cardiac remodeling [78–80]. In a canine model of ischemia and reperfusion, however, no effect of colchicine on infarct size was shown [81].

To translate findings to the clinic, it is of pivotal importance that these results are investigated and reproduced in highly translational large animal models with a similar anatomy, physiology, metabolism, and immune system to humans. To date, one study showed that pharmacological NLRP3 inflammasome inhibition with MCC950 was effective in reducing infarct size, and it led to the preservation of cardiac function in a dose-dependent way in a pig model of MI [82].

Besides beneficial effects of NLRP3 inflammasome inhibition in preclinical studies, contradictory results have also been reported. Sandanger et al. observed larger infarcts in NLRP3-deficient mice when compared with wild type mice [83]. They also observed that ASC deficient mice did not show a reduction in infarct-size. Additional analysis regarding inflammatory cytokines, chemokines, as well as cardiac immune cell infiltration, suggests that the absence of NLRP3 or ASC could remove cardioprotective signaling, since the possibility to perform TLR2-induced preconditioning was absent.

Jong et al. concluded that the NLRP3 protein does not play a role in the acute development of MI due to low cardiac expression [84]. A closed-chest mice model of MI was compared with an open-chest model to investigate whether surgical trauma by itself could contribute to the activation of NLRP3. NLRP3 was indeed detected in the hearts of the open-chest model but not in the closed-chest hearts. Thus, these results suggest that NLRP3 activation is mainly driven by surgical trauma and not due to cardiac I/R.

Recently, Zuurbier reviewed the role of the NLRP3 inflammasome in cardioprotective signaling showing that there is not only evidence that this complex is detrimental after MI but can also be involved in several pathways that are cardioprotective [85].

Interactions between the NLRP3 inflammasome and known cardioprotective signal molecules, such as STAT3 and Akt, have been observed in several preclinical studies where especially NLRP3 was involved in defective Akt and STAT3 signaling in the heart of mouse models of myocardial I/R injury [86].

Collectively, the results discussed above show the potential of NLRP3 inhibition in I/R injury after experimental MI but also provide evidence that warrants further preclinical research in closed chest, translational large animal models.

## Clinical Trials Targeting IL-1 After Acute MI

Two clinical pilot studies, focusing on treatment with anakinra, have been performed to investigate inhibition of NLRP3 inflammasome related inflammation post-acute MI (Table 1).

Anakinra is a human recombinant IL-1 receptor antagonist, targeting both IL-1β and IL-1α.

Patients with an acute MI were randomly assigned to anakinra 100 mg/day for 14 days or placebo in a double blinded fashion to test the safety and effects of IL-1 blockade on remodeling of the left ventricle (LV). In a pooled analysis of the two studies, anakinra showed a significantly lower increase in C-reactive protein between admission and after 3 days (+ 0.8 mg/dl, interquartile range − 6.4 to + 4.2, vs. + 21.1 mg/dl, interquartile range + 8.7 to + 36.6, *P* = 0.002). It did not show an effect on LV end-systolic volume index, LV end-diastolic volume index, or LV ejection fraction but anakinra treatment led to a lower incidence of heart failure within a timeframe of 12 weeks (placebo 30% vs. anakinra 5%, *P* = 0.035) [58, 59].

Anakinra is now further studied in a larger trial in patients with STEMI but the results have not been published yet [87].

## Conclusions

In the last 2 decades, multiple studies unveiled evidence that the NLRP3 inflammasome has a role in the pathogenesis of atherosclerosis, coronary artery disease, and myocardial ischemia reperfusion injury. Clinical trials like LoDoCo, CANTOS, and COLCOT showed that targeting inflammatory pathways related to the NLRP3 inflammasome in patients with established coronary artery disease can be beneficial in the prevention of future cardiovascular events.

In addition, preclinical studies highlight the role of the NLRP3 inflammasome in ischemia reperfusion injury after an acute myocardial infarction, but contradictory results have been reported as well.

The NLRP3 inflammasome and downstream cytokines are emerging as important potential targets for cardiovascular disease. Specific targeting of intracellular NLRP3 inflammasome proteins has not been investigated in clinical trials yet. Expanding translational research with selective NLRP3 inhibitors is necessary to fully evaluate the potential of NLRP3 inflammasome inhibition in cardiovascular disease.
